# Orchestrating differential data access for translational research: a pilot implementation

**DOI:** 10.1186/s12911-017-0424-6

**Published:** 2017-03-23

**Authors:** Marco Brandizi, Olga Melnichuk, Raffael Bild, Florian Kohlmayer, Benedicto Rodriguez-Castro, Helmut Spengler, Klaus A. Kuhn, Wolfgang Kuchinke, Christian Ohmann, Timo Mustonen, Mikael Linden, Tommi Nyrönen, Ilkka Lappalainen, Alvis Brazma, Ugis Sarkans

**Affiliations:** 1 European Bioinformatics Institute (EMBL-EBI), Wellcome Trust Genome Campus, Hinxton, Cambridge CB10 1SD United Kingdom; 2 Chair of Medical Informatics, Institute of Medical Statistics and Epidemiology, University Medical Center rechts der Isar, Technical University of Munich, Munich, Germany; 3 Heinrich-Heine Universität Düsseldorf, Coordination Centre for Clinical Trials, Düsseldorf, Germany; 4 European Clinical Research Infrastructure Network (ECRIN), Düsseldorf, Germany; 5 CSC - IT Center for Science Ltd, Espoo, Finland

**Keywords:** Data Access, Translational Research, Clinical Data, Biomedical Data, Health Data Protection

## Abstract

**Background:**

Translational researchers need robust IT solutions to access a range of data types, varying from public data sets to pseudonymised patient information with restricted access, provided on a case by case basis. The reason for this complication is that managing access policies to sensitive human data must consider issues of data confidentiality, identifiability, extent of consent, and data usage agreements. All these ethical, social and legal aspects must be incorporated into a differential management of restricted access to sensitive data.

**Methods:**

In this paper we present a pilot system that uses several common open source software components in a novel combination to coordinate access to heterogeneous biomedical data repositories containing open data (open access) as well as sensitive data (restricted access) in the domain of biobanking and biosample research. Our approach is based on a digital identity federation and software to manage resource access entitlements.

**Results:**

Open source software components were assembled and configured in such a way that they allow for different ways of restricted access according to the protection needs of the data. We have tested the resulting pilot infrastructure and assessed its performance, feasibility and reproducibility.

**Conclusions:**

Common open source software components are sufficient to allow for the creation of a secure system for differential access to sensitive data. The implementation of this system is exemplary for researchers facing similar requirements for restricted access data. Here we report experience and lessons learnt of our pilot implementation, which may be useful for similar use cases. Furthermore, we discuss possible extensions for more complex scenarios.

## Background

Translational research is a promising approach to speed up discovery of new therapies and diagnostic methods. In order to realise such objective, tight collaboration of biomedical researchers and clinical practitioners is required [1, 2]. Their work is data intensive [3, 4] and must rely on information technology to enable efficient data exchange and analysis [5, 6]. Compared to more traditional drug research, access to a larger variety of trials from diverse sources can improve the characterisation of benefits and unwanted effects of drugs and therapies at lower costs and better efficiency [7]. Drug approval processes and drug safety/effectiveness surveillance are improved by faster access to data about active ingredients similar to the ones being under consideration. An example of that is the effectiveness of using existing evidence, or even the prior obligation to make trial outcomes publicly available, to prevent selective reporting [8, 9], that is, the presentation of evidence that is favourable for the interest of the reporter (such as having a drug approved), and the exclusion of unfavourable evidence. Another potential advantages of these approaches is making clinical experimentation more efficient and avoiding the exposure of trial’s potential participant to known risks, as well as, for instance in the case that evidence shows adverse effects to particular health conditions, avoiding unnecessary risks. Ability to perform data analyses other than those for which clinical trials were originally conducted is another opportunity that clinical data sharing offers [10], which is relevant in the translational research field, enabling approaches like comparative genomics [11, 12]. Overall, this has social benefits such as faster improvement of healthcare and its safety, and increasing the confidence of the general public in the scientific community, public services and industry [10]. On the other hand, dealing with biomedical information, and with human patient data in particular, poses complex challenges with respect to ethical, legal and social implications (ELSI [13, 14]), which need to be addressed when software products are developed and IT infrastructures deployed [15, 16]. An obvious example is the wish and right of patients to keep their health information private, which can be motivated by various reasons, including the kind of relationship that an individual wants to maintain with his relatives and social relations [17, 18], the social stigma associated to certain diseases [19, 20], and access to private healthcare [21]. Another reason to resist data sharing lies in the commercial or academic interests of researchers, including the willingness to be the first to submit unpublished research, and the wish to produce evidence useful to file patent applications [22, 23]. These issues pose potential conflicts with the research needs. For instance, anonymization and reidentification-prevention techniques, which are used to grant data access while ensuring patient privacy, imply that data essential for a research goal might be concealed from the researchers [24, 25].

Life science shares technological challenges with other areas of science [26], and generic technological solutions can be employed, either of commercial or open source type [6, 27–29]. However, addressing ELSI in the translational research arena is particularly difficult, due to the above mentioned reasons, which can be summarised as heterogeneity of information systems, different types of professional roles involved, the conflicting needs to share information and, at the same time, ensure this is done in a way that respects patients and associated legislation [30–32]. The domain of biobanking and biosample research is characterised by special restrictive sample and data usage conditions, since highest ethical standards to ensure the support and participation of human research participants are required. In addition to confidentiality, consent about the data usage, intellectual property and data/sample ownership must be considered. Sophisticated mechanisms to provide restricted access to sensitive data is a way to address this problem. The risk of improper use of the data can be mitigated through legally binding agreements, subscribed by trial participants and researchers, which constrain the purpose for which data access is granted. Access is mediated by some form of a data access agreement between a data consumer and a data provider. These access agreements have to take into account legal and ethical requirements, professional guidance, and good practices. Agreements are in general executed by data stewards or data access committees, but recently they are implemented in electronic form employing software for identity and access management. This approach is not without difficulties, such as the impossibility to foresee useful research goals at the time of data and consensus collection [9, 10]. However, it can be seen as a compromise between the different needs that it addresses.

In this paper we report on a pilot implementation (from now on, ‘the pilot’) that aims at integrating research resources and clinical resources, including data bound to a varying range of access policies, from fully open to data requiring access approval. Implemented in the context of the BioMedBridges project [33–35], the pilot shows how identity and permissions management can be simplified by means of a modular approach, utilizing well known software components.

### The BioMedBridges project

The European Strategy Forum on Research Infrastructures (ESFRI) initiative has been promoting an agenda to build Research Infrastructures (RIs) in Europe since 2002 [36]. Its current agenda comprises 21 projects in all scientific fields. This includes RIs for the life science area, several of which teamed up in the FP7 BioMedBridges project. The main aim of this project was to facilitate the translation of ideas into medical applications, by promoting data interoperability in a variety of disciplines, across different scales. The project concentrated on five use cases, including cross-species data integration, personalised medicine, imaging, and structural biology. This work was supported by technological, cross-domain activities, such as terminology and data standards harmonisation [37], and secure access to data. The latter was investigated both from the point of view of ELSI, as well as what concerns the realisation of concrete IT solutions. All reports of the project are available [38]. The pilot presented here is documented in detail in the report D5.4 [39], which was preceded by the analysis and design done for D5.3 [40] and by the preparatory investigations on ELSI topics in D5.1 [40, 41] and D5.2 [42].

## Methods

In the following we describe the software components that we have employed to deal with the use case addressed by the pilot.

### The EBI Biosamples Database

The Biosamples Database (BioSD [43, 44]) is a public repository focused on biological sample information, which is maintained by the European Bioinformatics Institute (EBI). Its rationale is to provide a single access point to the information about the bio-materials used in biological and/or medical research. The users of this resource can search for biomedical samples of interest (e.g., based on phenotypical characteristics), and then navigate to external resources for accessing the data generated on those sample (e.g., microarray data in ArrayExpress [45], proteomics data in PRIDE [46]). Among other benefits, BioSD can aid translational research, since summarised clinical trial data and other information on medical samples are a significant part of its contents. For instance, one can perform a search based on a disease (e.g., using the keyword ‘leukemia’) and find results related both to clinical research (e.g., the sample group ‘SAMEG158683’, concerning human patients and coming from the COSMIC repository [47]), and model organisms (e.g., the group ‘SAMEG22290’, linked to mouse transcription data in ArrayExpress).

### BBMRI Hub and biobanks

BBMRI (Biobanks and Biomolecular Resources Research Infrastructure) is a European research infrastructure [48]. The BBMRI-LPC (BBMRI - Large Prospective Cohorts) project [49] aims to build a network for large European prospective studies in order to facilitate transnational research about human health and diseases. The ‘LPC Catalogue’ [50], based on the MIABIS standard [51] and data warehouse techniques, provides a structured overview of the cohorts participating in the BBMRI-LPC project and supports researchers in gaining access to their biomaterials. For the purpose of the pilot we set up an adapted instance of the LPC Catalogue, the ‘BBMRI Hub’ [52]. It provides enhanced functionalities for access to detailed data, including information about individual human samples stored in external biobanks. Moreover, the Shibboleth and REMS systems (see below) were integrated in the hub, enabling identity management and access control.

### Resource Entitlement Management System

The Resource Entitlement Management System (REMS [53]) is an open source software that can be used to manage policies for granting access to resources, including digital data [54]. For instance, a data manager may establish that an application procedure is required to access clinical data from a web application like BBMRI Hub, and information about the purpose of the research, or approaches to data protection need to be provided by the applicant and approved by the Data Access Committee (DAC). REMS allows data managers to define per-resource authorisation workflows, which can be used by software systems to ensure users are entitled to see the data requested, and, if not, it facilitates the actions needed for the access to be granted. REMS centralises and simplifies procedures that are often bureaucratic and hard to keep track of. REMS can be integrated with Shibboleth (see the next section), both for the delegation of user authentication, and for the distribution of the entitlement attributes granted by the DACs to authorise access to protected data.

REMS is an mean to manage the agreements between multiple data owners, data sets and data users. As such, it flexibly delegates these responsibilities to DACs and to the contents of the data access agreements that REMS allow DAC members to define. This approach has been successfully used with the European Genome-phenome Archive, which of data access is based on REMS [55].

### Identity Management via Shibboleth

In an interconnected world, where multiple providers are able to serve integrated Internet applications and provide a uniform user experience across them, standardised approaches to manage digital identities are ever more important. The identity federation standard SAML is one of the most popular solutions of this kind. Open source implementations, such as Shibboleth [56], are available for many platforms and applications. Shibboleth offers relatively simple methods to wrap areas of a web application (e.g., via URL patterns), so that, before serving a web request, an unauthenticated user can be forwarded to a common login process, where (s)he can select an identity provider (IdP), such as the authentication system managed by their organisation. After authentication, Shibboleth creates a user session, filling it with identity attributes, which are sent back to the original request (via browser forwarding), where the application (acting as a service provider, SP) can check the existence of a session and the associated attributes. A single session can be shared by multiple SPs, thus allowing for centralised accounts and single sign-on. Moreover, SPs can enrich the Shibboleth session with their own user-specific attributes. This means that, in our pilot, REMS can send resource entitlements approved for a user to the resources needing to check for their existence. Based on the SAML standard [57], Shibboleth represents a flexible, standards-compliant solution to decouple application logic from application access and permissions management, delegating the latter to organisation-wide identity managers (e.g., institute’s account directory and management). This is usually arranged into identity federations, essentially sets of organisations, identity providers, and applications where there is mutual trust among the participating parties. This is relevant to the management of sensitive data, where, for example, policies might require specific forms of identity proofing, such as presenting a government-issued photo-ID document to a registration authority.

## Results

**Fig. 1:**
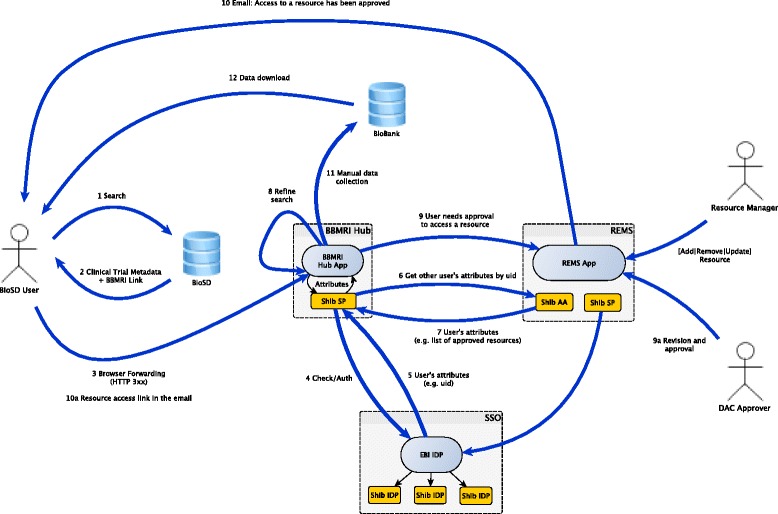
The workflow implemented for the BioMedBridges secure access pilot. Taken from [39]

Figure 1, taken from the previously mentioned BiomedBridges report [39], summarises the workflow that we have implemented in the pilot presented in this paper, based on previous work within the BiomedBridges project [40, 41]. This workflow supports the use case where a researcher is looking for samples of interest, both human and non-human, with an aim to explore experimental data derived from such samples, as well as acquire bio-material for further studies. As discussed above, EBI’s Biosamples Database is a significant starting point for such a use case. We uploaded summaries about demonstration data sets onto BioSD, linking them to more detailed information available in the BBMRI Hub. For instance, a search initiated from BioSD (step 1 in the figure) might lead (step 2) to the page about the data set named DE_Biobank7.^1^ The decision to use realistic demo data, which does not belong to real patients, allowed us to concentrate on the technical issues, leaving aside the legal and ethical implications.

The BBMRI Hub provides summary information for the requested dataset (e.g., number of samples for each biological characteristic), and mediates the access to individual level data (e.g., anonymised patient records). Namely, the Hub checks that the requesting client has an associated Shibboleth session (it uses the Shibboleth Java API, and the SP plug-in for the Apache web server). If this is not the case, the Shibboleth component transparently redirects the user to a sign-on page (step 4; we have set up a single sign-on demonstration service for the pilot). After successful authentication the browser is sent back to the Hub, where the now-existing user session is used to query REMS-related session attributes. These contain the list of resource entitlements associated to the user, such as the access rights the user has for the biobank (s)he is trying to access. Such information is obtained by coupling REMS with Shibboleth’s Attribute Authority component (steps 6/7). If the user does not have the rights to access the requested data, (s)he is forwarded to REMS (again, via browser redirection), to apply for such access (step 9), i.e., provide the research plan, confirm compliance with the data access agreement etc. The exact workflow for the resource access application depends on the resource being accessed, and REMS allows the data manager to define it. Once the user has completed his/her application, this is forwarded to a DAC user for approval. Approved applications (or rejections) are notified to the applicant via email messages, which are also used to send a link to resume the pilot workflow from step 11, i.e., access the protected biobank. A variant of this workflow can happen at step 8, when the initial searches can be refined in BBMRI.

### Systems robustness, testing and performance considerations

The pilot aims at demonstrating the technical feasibility to utilize the above workflow in order to mediate access to data of the LPC Catalogue which is reflected in the BioSD. As such, we have tested the infrastructure as a whole with an initial small set of 3 test Shibboleth federations and about 10 Shibboleth-authenticated users. In order to perform such tests, we have populated BioSD and the LPC demo catalogue with information about 14 fictitious human data sets.^2^ The components that the pilot is based on have been extensively tested, by both the original developers and the many organisations that use them. Both cases include the organisations participating in the pilot.

As an example, the Biosamples database has been developed following sound software engineering methodologies, including extensive use of test-driven coding an unit tests (for instance, [58, 59]). The repository currently stores about 5 million samples, grouped into about 58 thousand data sets, provided by 14 thousand organisations.^3^ From internal web server logs, we can estimate that BioSD successfully processes on average 158’000 requests per day from about 28 unique client IP addresses, without significant traffic peaks. This makes it clear that BioSD is a valuable resource for biomedical samples, involving a significant number of users. We keep the user community engaged by running periodic exercises of user experience [60] and outreach activities [61]. Regarding the quality of data and annotations in BioSD, while this varies due to our aim to accommodate the needs of as many data submitters as possible, we host sample information from important and well known biomedical sources (see previous section), and we provide ontology-based semi-automatic data reannotation, which improve the original metadata [62].

As another example, at the time writing the LPC Catalogue contains data about 22 large population-based biobanks, including two European networks (MORGAM, EPIC), and data about more than 3.8 million biosamples comprising more than 9 material types (e.g. DNA, cDNA/RNA, whole blood, blood cell isolates, serum, plasma, tissues, cell lines, urine). Eleven out of the 22 participating biobanks state to have diseases of the circulatory system as a focus of research, followed by endocrine, nutritional, and metabolic diseases, mental and behavioural diseases, as well as diseases of the respiratory system with ten biobanks each, respectively. The Catalogue plays an important role in the context of calls for tender within BBMRI-LPC. Two out of four proposals which have already been approved specifically request biosamples from three participating LPC cohorts each. The other two proposals involve all participating LPC cohorts which comprise a sufficiently large number of cases with obtainable biosamples. Further proposals are under review. This indicates the practicability, importance and user acceptance of our approach [63].

In order to estimate the performance of an infrastructure like the pilot, consider the following. According to performance test results [64], a modestly performant server (single or dual-core recent CPUs, up to 12Gb RAM) can uphold 50–100 Shibboleth requests per second, under a workload of up to 3500 parallel requests. The same tests show that Shibboleth scalability is also good, with request latency staying below 5 s for a workload under 500 threads. In [26] it is shown that these limits are well compatible with what we would expect in a scenario like the one described in the pilot, including the figures about organisations managing up to 30’000 users per year through Shibboleth. The above-mentioned figures about BioSD web traffic are well below the values shown above about Shibboleth, especially considering that only a small part of BioSD traffic would come from authenticated users, interested in protected clinical data. The LPC Catalogue and REMS are also able to support similar workloads. These rough figures make us confident that the approach proposed by our pilot is usable from the performance point of view. Moreover, the results achieved in the BioMedBridges project will be leveraged by the CORBEL project, which aims at developing harmonised user access to biomedical data from research infrastructures [65], and by the ELIXIR-EXCELERATE [66] project, which will ease data access in the ELIXIR biomedical network [67]. CORBEL and EXCELERATE are involving 11 and 41 organisations across Europe [68, 69], for a total number of researchers in the order of hundreds. Again, these figures are well below the limits mentioned above.

A further point about robustness lies in the process in place to create credentials for users participating in an identity federation like the test federations that we have set up for the pilot. Clearly homogeneous and safe procedures must be established for that. While this is not within the scope of this paper, we wish to make a few considerations, based on the experience with our organisations and resources. Similarly to other cases [70], all the pilot participants have well defined security policies. For instance, EBI has an internal policy such that electronic credentials are supervised by a senior staff sponsor, including initial identity certification.

## Discussion

Common open source software components are sufficient to create a secure system for differential restricted access to sensitive data. The components used to implement the pilot are freely reusable to realise a similar infrastructure in a situation similar to the one we address. In particular, Shibboleth and REMS can be adapted to a different set of biomedical resources that other organisations might want to integrate. Technical details and first experiences about the pilot implementation are described in a report for the BioMedBridges project [39]. Such report includes implementation details that would be useful for the pilot reproducibility. In this section, we are discussing the feasibility, advantages and limitations of our approach, to provide additional information for researchers confronted with similar data protection needs when dealing with similar use cases, and explain our experience with the pilot implementation. Furthermore, we frame the pilot work into a wider legal and technological context.

### Addressing security and data protection issues

The work on the pilot was preceded by a preliminary assessment of the legal and ethical situation for data protection and data privacy involved in access and sharing of open data, together with sensitive data, as well as, on a more technical level, the security risks related to biomedical data exchange [40]. The STRIDE methodology [71] was applied to evaluate security threats. This consists of analysing well known threats that occur in software systems (Spoofing, Tampering, Repudiation, Information Disclosure and Elevation of privileges), assessing the extent to which they are present in the system under consideration, and establishing countermeasures to eliminate or reduce the impact of these threats. Additionally, we applied the LINDDUN methodology [40], which allows for further, privacy-specific threat assessment (considering the aspects of Linkability, Identifiability, Non-repudiation, Detectability, Disclosure of information, Content Unawareness, Policy and Consent Non-compliance). We reviewed the threats to security and privacy that the pilot (or similar solutions) is able to address (Table 1), and here we analyse the advantages that our approach might offer in similar situations.

**Table 1:** How STRIDE threats are addressed in the pilot (or could be in similar scenario)

STRIDE Threat/ Function	Shibboleth/Id Federation	REMS	Domain Apps (BioSD, BBMRI Hub, more)	Infrastructure (eg, web servers, network)
Spoofing/Authenticity	Authentication HTTPS/TLS/X.509 Limit distributed attributes Proper Software Engineering (PSE)	Limit distributed attributes PSE	PSE	- HTTPS/TLS/ X.509 - PSE
Repudiation/Accountability	Authentication Logging (must be law-compliant, eg max retention time)	Logging PSE	- Logging	- Logging
Info Disclosure/Confidentiality	HTTPS/TLS/X.509	- Subscribed policies (no data out of Id Federation) HTTPS/TLS/X.509	HTTPS/TLS/X.509	HTTPS/TLS/X.509
DoS/ Availability	- PSE	- PSE	- PSE	- Redundancy - Firewalls - PSE
Elevation of Privileges/Authorisation	- Only required attributes distributed - PSE	- Only required attributes distributed - PSE	- PSE	- PSE

PSE refers to software design and testing, best practices, established methodologies, techniques and frameworks. As for the biomedical-specific risks identified by the LINDUN methodology, REMS policies help with facing all those risks, as it does the security and reliability of the pilot software components

The combination of Shibboleth and REMS limits the risk that confidential information is disclosed to unauthorised persons, by means of simplification and centralisation. REMS helps in ensuring that data access authorisations are granted according to the legal requirements associated with the data sets (e.g., the kind of consensus the patients have given). Moreover, the activity tracking functionality provided by these two tools offers a basis for keeping evidence of compliance with law and regulations, and holding users and data managers accountable for their actions. The underlying SAML standard of Shibboleth mitigates spoofing threats (i.e., pretending to be someone else or faking invalid credentials) by exploiting standard and reliable technologies, such as encryption and digital signature of SAML messages [72] based on certificates following the X.509 standard [73], or the HTTPS protocol coupled with the TLS layer [74]. Restricting the session attributes that are distributed via Shibboleth can reinforce the protection against spoofing. It is important that the participating applications use the same security protocols (as BBMRI Hub, REMS and BioSD do). More general countermeasures have been applied to the pilot’s underlying infrastructure. For instance, redundant web server architectures and firewall-based IP-filtering have been deployed to both ensure reliability of the services and minimise the risk of denial-of-service attacks (DoS [75]). Using logging facilities and making authentication mandatory allows for user accountability and non-repudiation (this must be done according to the local laws, e.g., ensuring periodic deletion of older entries). Adopting the best software engineering practices is another general precaution that limits security and privacy risks. For example, application configurations are carefully managed, so that unsafe settings (e.g., too liberal access rights, clear-text passwords) do not compromise security or data protection. As another example, prevention of code injection attacks [76] and thorough testing [77, 78] give reasonable protection against many threats.

Regarding the threats identified by the LINDDUN approach, while the pilot doesn’t address data anonymisation and identifiability issues, this is delegated to the data management policies defined at the level of local biobanks, the managers of which are responsible for collecting informed consent documentation from patients. This is eased by the functionality available in REMS, which a data access committee can use to match the consent given by the patient to specific data uses according to the researcher commitments.

Having components grouped in an identity federation improves user reliability, thanks to the fact that their identities are verified by mutually trusted organisations. Further restrictions would be possible, such as preventing data from leaving a given IT network (hardware-level encryption [79] could be one way to realise it).

As mentioned above, data access agreements helps in balancing the advances that are achievable from access to a wealth of biological data data with the ELSI needs. Because of that, it is important that the process of granting access to data and monitoring the access once it is approved rely on efficient and seamless tools. The approach used by the pilot has several advantages: the kind of formal commitment that one needs to use a data set is clearly associated to the data: the electronic management of the interaction between DACs and requestors is more efficient than the exchange of physical paperwork and makes it easier to keep track of who has given access to who, what and for which purpose. In turn, this facilitates the procedures to make data accessors accountable for their activities. Moreover, one can leverage systems like REMS to improve the circulation of data access approval processes, including the conditions upon which such approval is granted. This can extend to trial participants and the general public, which, in turn, can improve accountability of and trust in the scientific community, often an important point in collaborating with the public for the purpose of obtaining study data.

While not in the scope of this paper, it is worth to mention that the adoption of systems similar to the pilot has an economical impact on the management of research and clinical practice. In fact, using open source components, some of which might already be in place in many organisations, has the potential for cost reduction, as well as better control over the technology that is being deployed. Furthermore, signing data use agreements to clarify commercial aspects in the early stages of a research activity can prevent disputes over the right of researchers to publish results based on third party data, or the interest in exploiting data for patenting and regulatory purposes.

### The problem of common released attributes

A well known, mostly social problem in digital identity management is the need for every organisation participating in an identity federation to agree on a common set of attributes that their IdPs should release, so that the applications (SPs) can verify a user and the associated entitlements to resources (as in the case of REMS entitlements). The general scenario is such that each of the N IdPs in the federation must distribute the attributes required by every of the M SPs, with a different set of attributes for each of the N*M pairs, and a corresponding number of negotiations, documents to sign, etc. While we did not have many parties in the pilot, we suggest to deal with this problem in a production-grade infrastructure by means of a proxy agent between IdPs and SPs. The proxy behaves as a SP for IdPs and as an IdP for the SPs. This way only M negotiations have to be made on the attributes needed by SPs, and further N agreements are made with the IdPs, regarding which attributes they will release to the (trusted) proxy. This simplifies and reduces the problem to the order of N + M. The ELIXIR-EXCELERATE project [66] is deploying a solution based on this approach. It is worth to mention that we have not addressed other related issues, for instance: a) the fact that organisations tend to be conservative on the release of user attributes, due to concerns about the personal information laws [80], b) the lack of standardisation in attribute names and semantics [81]. The proxy approach would help mitigate the impact of such issues, by uniforming the attributes that the proxy releases, and by helping the participating organisations with dealing with the legal issues.

### Related work

Many different approaches and systems are used for tackling the aims and issues we have addressed in the pilot. Biological material repositories similar to BioSD exist, varying in scope [82, 83], geographical reference area [84] and scale [85, 86]. BioSD is mainly a European reference resource for public biosample data and metadata. A similar variety exists in the arena of clinical data resources [87]. In this field, the LPC Catalogue is among the most prominent biobank catalogues in Europe, while a wide range of biobanks with different scales and scopes exist [88]. Several technologies and approaches are available to manage identities and application access rights [27–32]. For instance, commercial systems like OpenID [89] tend to prefer technical simplicity over advanced features (e.g., identity federation is not a standard feature within OpenID). We have chosen Shibboleth for multiple reasons: it is reliable software based on the SAML standard, it is well-known among research organisations, and the organisations involved in the pilot were already using Shibboleth when we started our work. Permission and access management is an issue wider than technology, which encompasses IT solutions, policies like access audits and new personnel checking and regulatory compliance [16, 90] The access control used in REMS can be seen as a variant of a lists-based access control approach (ACL [91]). Compared to similar products [92–94], REMS is focused on granting resource access based on the commitment to a data access agreement, and the final approval from personnel with the data access control role. Moreover, REMS allows for the definition of workflows to obtain and finalise the access approval procedures, and it logs the actions during the execution of these workflows. Finally, both REMS and the other components we have used are modular and can be composed into a larger system (e.g., with respect to the distribution of identities). While one might prefer simpler options on a smaller scale [95], our approach gives the flexibility to implement larger infrastructures with existing common technologies. The approach used in the pilot does not address the further data protection that is often ensured by establishing different data access levels (e.g., original patient records, de-identified/obfuscated data, aggregated data, disclosure of only summary statistics, computed at the source of data [96]) and by classifying users based on user trustworthiness [97, 98]. The pilot approach is agnostic with respect to the resource that is controlled and the specific protection mechanism that this has in place, which is made possible by the fact that both Shibboleth and REMS essentially see a resource as a reference, such as a URL to a web application or a web link to a file download. For instance, one might adopt our approach for mediating access to resources providing data summaries in ways similar to the BBMRI [98, 99], as well as in case of resources that grant access to web services [100] and local computations [96].

#### The pilot in the context of data access frameworks

In life science increasingly medical data have to be effectively accessed and linked. This expanding volume of human data is stored in various databases, repositories, and patient registries, while protecting data privacy and the legitimate interests of patients as well as data subjects. Regarding the purpose of ensuring protection of human data while enabling data sharing, several approaches have been suggested that range from the creation of a political framework in the form of resolutions or treaties, to operational guidelines for data sharing [101]. Such frameworks include concepts like legitimate public health purpose, minimum information necessary, privacy and security standards, data use agreements [102], ethical codes like the IMIA (International Medical Informatics Association) Code of Ethics for Health Information Professionals [103] and AMIA’s (American Medical Informatics Association) Code of Professional and Ethical Conduct, guidance for genomic data, and potential privacy risks [104]. More concrete approaches are a human rights-based system for an international code of conduct for genomic and clinical data sharing [105], recommendations about clinical databases and privacy protections [106], and healthcare privacy protection based on differential privacy-preserving methods (iDASH, integrating Data for Analysis, Anonymization, and Sharing) [107, 108].

Genetic sequence databases are an important part of many biomedical research efforts and contained in many data repositories and biosamples databases. However, human genetic data should only be made available if it can be protected so that the privacy of data subjects is not revealed. The problem is that individual genomic sequence data (e.g. SNPs) are potentially “identifiable” using common identifiers [106, 109, 110]. In biobanking many new population biobanks and cohort studies were created to produce information about possible associations between genotype and phenotype, an association that is important to understand the causes of diseases. Together with BBMRI, different initiatives exist that address the protection of data privacy and that further the standardization and harmonization of data management of genomic data and the sharing of data and biosamples, for example: Public Population Project in Genomics (P3G [111]), International Society for Biological and Environmental Repositories (ISBER [112]), Biobank Standardisation and Harmonisation for Research Excellence projects [113] and the Electronic Medical Records and Genomics (eMERGE) Network [11, 114].

The constraints arising from limitations defined by the informed consent of the data subject have to be reflected in data access agreements and data transfer agreements. In general, the rule applies that data can only be made available to the extent that is allowed under the local legal requirements relevant for the data provider including ethics votes, vote by data access committee and the consent by the data subject. Data sharing should be an important part of an overall data management plan, which is a key element to support data access and sustainability. A data sharing agreement should supplement and not supplant the data management plan because the sharing agreement is about relationship building and trust building. It supports the long term planning and finding ways to maximize the use of data.

Anonymisation is becoming increasingly more difficult to achieve due to the increase in health data such as genomic data that is potentially identifying. As mentioned above, although anonymisation is protecting the privacy needs of the data subjects, it is an imperfect solution and must be supplemented by additional solutions that build trust and prevent researchers from trying to identify study subjects. In the end, what is necessary for research is a culture of responsibility and data governance when dealing with human data. Building blocks that support and strengthen such culture are data sharing agreements, strict authentication and authorisation methods and the monitoring and tracking of data usage. The created pilot fits into such efforts, because, by using and combining several open source components, it created an efficient authentication and authorisation framework for the access to sensitive data that can support efforts for trust building. The pilot must be seen in connection with the creation of a European Open Science Cloud, a federated environment for scientific data sharing and reuse, based on existing and emerging elements [115]. The complexity of current data sharing practices requires new mechanisms that are more flexible and adjustable and are employing proven components, like the open source authentication components of the pilot.

### Possible future developments

#### Ethical, legal and social implications

As already mentioned above, ethical, legal and social implications (ELSI) are of utmost importance when dealing with management of human health data [13–16]. The BioMedBridges project has extensively worked on such issues and the associated software tools [39–42]. In a scenario like the one presented above, there are several components and processes where such tools could be integrated. To guide researchers with no extensive legal knowledge through the relevant legal requirements, the Legal Assessment Tool (LAT) was developed in BioMedBridges [116]. LAT provides researchers with an online, interactive selection process to characterise the involved types of data and databases and provides suitable requirements and recommendations for concrete data access and sharing situations. Links to the LAT [117] were added to the BBMRI Hub, in order to guide data managers when assessing the data sharing policies that should be adopted for the data sets managed in the system, as well as the implications of granting access to them. The Human Sample Exchange Regulation Navigator (hSERN), a web resource about legal aspects involved in exchanging human information [118], is another resource that would be useful to both biobank providers and REMS users having the DAC role. The BBMRI Legal Wiki [119] is a similar resource with an EU perspective, which could be useful when data need to be exchanged across EU member countries. The International Policy interoperability and data Access Clearinghouse (IPAC [120]), a tool serving information about policy interoperability on international level, could provide more direct help to the members of DACs who need to craft forms within REMS to be presented to users applying for dataset access. In fact, IPAC contains form templates reusable for such a purpose. Data sharing for research purposes must be opened for human health data, and these tools are means to clarify the conditions for data sharing. They can complement our pilot implementation of a system for restricted data access, providing each components with appropriate safeguards, restrictions and responsibilities and in this way supporting a culture of responsibility and data governance for the sharing of human data.

#### Batch and programmatic access to data

The pilot focuses on software components that interact with users via the web. Extensions for programmatic access that are inspired by our solution could be implemented relatively easily. For instance, if an approved user needs to access the BBMRI Hub from an application or a web service that (s)he is running (or such access is triggered by the main web application the user is interacting with), this component would work like a web browser, forwarding IdP and REMS-related requests. Shibboleth has sample implementations [121] to make a web service aware of the fact that a data request might return an IdP-related link and needs to be forwarded elsewhere. Unattended batch processing programs would work in a similar way, although they would likely need security reinforcement, for example by means of time-limited authentication tokens and key pairs [12]. A particular type of web service is represented by SPARQL endpoints to serve linked data [122–124], which are increasingly important in life sciences, and which have been widely studied in BioMedBridges [37, 125]. In addition to considering this type of access just like any other web service and thus applying what we have outlined above, it would be worthwhile to consider more specific approaches, which analyse SPARQL queries to decide how to dispatch them across protected data repositories, on the basis of defined access policies [126–128]. In the context of the pilot, such access policies could be provided to a federated query engine by integrating it with Shibboleth and REMS. As a final note, one should take into account the impact of these techniques on ELSI, e.g., it might be the case that the law requires explicit consent for sending data to a third party component, such as a web service.

## Conclusion

Protected access to digital resources related to translational research is a significant challenge that encompasses technology, law, ethics and society, and the importance of this for translational research is growing. In the work presented we have shown an approach to face this challenge, based on open, common components and standards. In addition to showing the feasibility of such an approach, our pilot for secure biomedical data access can be a reference for similar data access use cases and can offer useful experience and lessons learned for researchers confronted with similar data protection needs. Moreover, it encourages researchers to use open source components as the basis to integrate ELSI tools into data management software, as well as for developing more complex usage scenarios, such as data access based on web services and linked data technologies. The pilot can be a model for researchers who want to use readily available open source modules to create a solution for the handling of sensitive data. Compared to other solutions, our approach is simple yet effective, being focused on the authentication and authorisation problem, without dealing with the technical about the access to specific resources. This also means it is relatively easy to realise a pilot-like solution over components already in place (only some integration work is needed between those, Shibboleth and REMS, existing Shibboleth-based IdPs can be reused) and it is not required to deploy a new infrastructure, as it is the case for SHRINE [99] and i2b2-based systems [6, 98], caGRID [93] and DataSHIELD [96]. At the same time we delegate to systems like Honest Broker [100] advanced privacy enhancing techniques. The work between the research infrastructures that have participated in the pilot is continuing in CORBEL [65] and AARC [129] projects. In particular, similar production services are being implemented thanks to the funding provided by the EXCELERATE initiative.

## Abbreviations

BBMRI: Biobanks and Biomolecular Resources Research Infrastructure, related to the BBMRI-LPC project and LPC Catalogue (also named BBMRI Hub), see the main text
BioSD: European Biosamples Database (see the main text)
DAC: Data Access Commitee (see the main text and also: REMS). Institutional Review Board (IRB) is a commonly used term that represents a similar concept
Data de-identification The process of deriving more generic data from detailed sensitive and individual human data: e.g., through the removal of identifying information (name, address), by means of ranges (age 0–5 in place of precise birth dates), by means of aggregation (the number of patients aged 0–5, rather than individual records)
ELSI: Ethical, Legal and Social Implications. The kind of issues arising when dealing with human biomedical data
HTTPS: SSL, TLS, X.509, HTTPS refers to the World Wide Web protocol wrapped by some communication channel protection/encryption technology. TLS (Transport Layer Security) is a typical protocol to do so, which succeeded SSL (Secure Socket Layer). These protocols use the standard X.509 to deal with data encryption (i.e., to encode data in a way that they are only readable by a target recipient and, in the X.509 context, with the certainty of the data producer identity)
IdP: Identity Provider, a system like Shibboleth, used to manage user information and authentication in a network of organisations and IT applications
LINDDUN: Linkability, Identifiability, Non-repudiation, Detectability, Disclosure of information, the specific privacy-related threats addressed by the LINDDUN methodology to secure an IT system to access sensitive human data
REMS: Resource Entitlement Management System, which enables Data Access Commiteess (DAC) to administrate access to data resources (see the main text)
SAML: Security Assertion Markup Language, a standard XML-based format for exchanging authentication and authorization information between IT components. Used by the Shibboleth system (see)
Shibboleth: An identity management system based on the SAML standard (see the main text)
SP: Service Provider, an application that relies on an IdP (see) to manage user authentication and authorization. In the specific context of Shibboleth, a programming library that allows an application developer to realise SP functionality managed via Shibboleth
STRIDE: Spoofing, Tampering, Repudiation, Information Disclosure and Elevation of privileges, the threats addressed by the STRIDE methodology to secure an IT system

## Footnotes

1
http://www.ebi.ac.uk/biosamples/group/SAMEG299071. The list of all the data set records uploaded for the pilot are available at https://goo.gl/SVXZ3A.

2 Ibid.

3 This can be computed by means of the RDF/SPARQL endpoint, e.g., https://goo.gl/ti4S34
